# Resveratrol acts via melanoma-associated antigen A12 (MAGEA12)/protein kinase B (Akt) signaling to inhibit the proliferation of oral squamous cell carcinoma cells

**DOI:** 10.1080/21655979.2021.1934242

**Published:** 2021-06-04

**Authors:** Yu Shang, Yu-Ling Jiang, Li-Jun Ye, Li-Na Chen, Yue Ke

**Affiliations:** a Department of Stomatology, Xiangyang No.1 People’s Hospital, Hubei University of Medicine, Xiangyang, Hubei Province, P.R.C; b Department of Stomatology, Xiangyang Central Hospital, Affiliated Hospital of Hubei University of Arts and Science, Xiangyang, Hubei Province, P.R.C

**Keywords:** Resveratrol, MAGEA12, oral squamous cell carcinoma cells, proliferation

## Abstract

The present study examined how resveratrol affects cell growth and MAGEA12/Akt signaling pathway in OSCC cells. Cal-27 cells were transiently transfected with a plasmid encoding *MAGEA12*, and the effects of overexpression were assessed in terms of cell viability, colony formation and the epithelial–mesenchymal transition. Cal-27 cells and *MAGEA12*-overexpressing cells were treated with resveratrol, then the cell viability and colony formation were also assessed by CCK8 assay and microscope, respectively. Levels of *MAGEA12, p-Akt, Akt, Cyclin D1, and CDK14* genes and these proteins were analyzed using quantitative reverse-transcription polymerase-chain reaction and western blot. In the present research, we first generated and transiently transfected *MAGEA12* plasmid into Cal-27 cells. Our results suggested that overexpressing MAGEA12 led to an increase in levels of phospho-Akt, which was associated with increased cell viability, colony formation. Moreover, overexpressing MAGEA12 also resulted in the up-regulation of Cyclin D1 and CDK14, indicating MAGEA12 induces the cell proliferation of Cal-27 cells. In addition, these effects were partially reversed by inhibiting Akt. Furthermore, resveratrol could inhibit the proliferation and colony in Cal-27 cells and decrease the expressions of MAGEA12 and p-Akt depending on the time and concentration. These effects were also partially reversed by MAGEA12 overexpression and Akt activation. In summary, resveratrol may suppress the growth of OSCC cells by inactivating MAGEA12/Akt signaling. These findings suggest that resveratrol may be a therapeutic drug for OSCC in clinical.

## GRAPHICAL ABSTRACT

**Figure:**
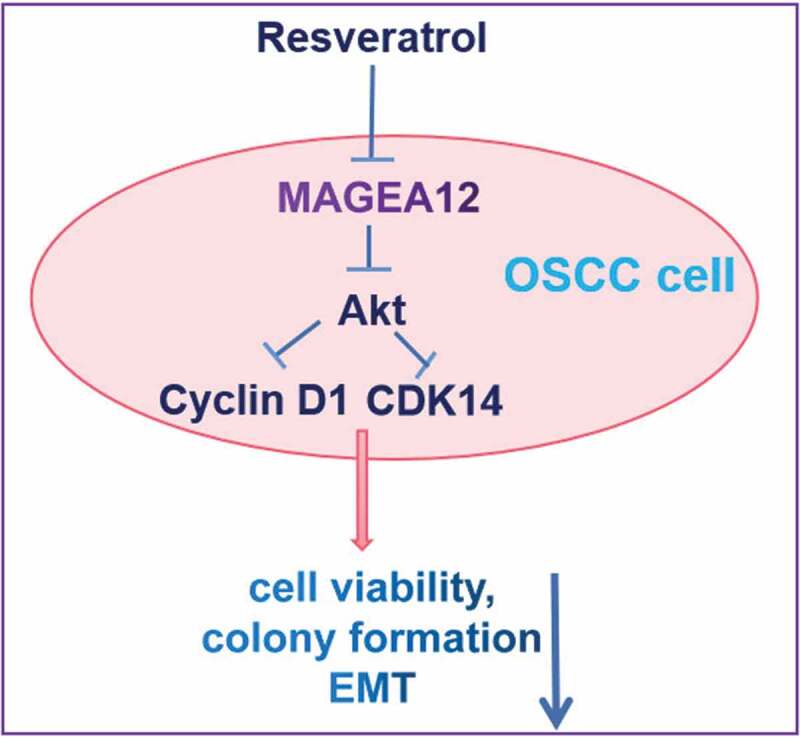

## Introduction

Oral squamous cell carcinoma (OSCC) accounts for about 90% of cases of oral cancer [1]. Surgery is the standard treatment for OSCC in early stages, while effective treatments for advanced disease are lacking [2]. Some genetic risk factors have been identified for OSCC [2], but little is known about the molecular mechanisms behind the disease, which hampers efforts at early diagnosis and treatment [3].

One protein potentially involved in OSCC is melanoma-associated antigen gene A12 (MAGEA12) [4], which belongs to the MAGEA family of antigens first identified in tumors of the testis [5,6]. MAGEA12 down-regulates tumor suppressor p21 and thereby promotes proliferation of primary prostatic carcinoma [7], and it also appears to act as an oncoprotein in other types of cancer [6–8]. How MAGEA12 may promote tumor progression in cancers such as OSCC is unclear.

Akt signaling is closely associated with tumor cell proliferation, migration, and invasion [9–12]. For example, activating Akt signaling promotes progression of cutaneous squamous cell carcinoma [13,14]. Furthermore, extensive observations have demonstrated that Akt is involved in the occurrence and development of OSCC [15–17]. It is well known that Akt is closely related to the proliferation, migration, apoptosis, and invasion of OSCC cells *in vitro* [16]. Additionally, it is well known that G1 progression-related gene (*Cyclin D1*) and mitotic-related gene (*CDK14*) contribute to cell cycle progression and maintain cells in a highly proliferative status [17]. Furthermore, Akt signaling also regulates the expressions of Cyclin D1 and CDK14 to mediate the proliferation of cancer cells [18]. The association of MAGEA12 with Akt in OSCC remains poorly understood, and whether MAGEA12 promotes the proliferation and EMT of OSCC cells via activating Akt is unclear.

Resveratrol (Res) is a natural phytoalexin product, and is widely distributed in a variety of plants. As an important component of red wine, resveratrol shows efficiency effects on antitumor properties [19]. It also reported that resveratrol inhibits proliferation and migration through Akt signaling in hepatocellular carcinoma cells [19]. Resveratrol has also been reported to inhibit the proliferation of pulmonary arterial smooth muscle cells by regulating Akt/Cyclin D1 signaling [20]. Whereas whether resveratrol suppresses the cell proliferation of OSCC cells and inactivates Akt/Cyclin D1 and CDK14 signaling pathways via inhibiting MAGEA12 is still unclear.

Therefore, we supposed that resveratrol could decrease the growth of OSCC cells by suppressing MAGEA12/Akt signaling pathway. To begin to identify its oncogenic mechanisms of action, the present study examined the effects of resveratrol on viability and proliferation of OSCC cells, as well as the potential role of MAGEA12/Akt in mediating those effects.

## Material and methods

### Cell culture and treatment

The human OSCC cell line Cal-27 was obtained from Beijing Zhongyuan (Beijing, China) and cultured in Roswell Park Memorial Institute (RPMI) 1640 medium (HyClone, Waltham, MA, USA) supplemented with 10% fetal bovine serum (HyClone) and 1% streptomycin (100 U/mL) and penicillin (100 U/mL) at 37 °C in a humidified incubator with 5% CO_2_. In some experiments, cells were also incubated with the Akt inhibitor MK-2206 (Sigma, USA), Akt agonist SC79 (Sigma, USA) and resveratrol (Sigma, USA). The gradient concentrations of resveratrol (0, 10, 20, 50, and 100 μM) were added into cells for different time points. The test in each group was repeated three times.

### MAGEA12 overexpression

The *MAGEA12* overexpression assay was performed as previous report [21]. Briefly, a plasmid encoding human *MAGEA12* and empty vector (control group) were designed and generated by Shanghai Gene Pharma (Shanghai, China), and Cal-27 cells were transiently transfected for 4 h with the plasmid using Lipo2000 (Invitrogen, Waltham, MA, USA) according to the manufacturer’s instructions. Then, the transfection medium was exchanged with fresh medium and subsequent experiments were performed as described below.

### Assay of MAGEA12 mRNA levels

Total RNA was extracted from cells using Trizol (Invitrogen, Waltham, MA, USA) according to the manufacturer’s instructions, and relative levels of *MAGEA12* mRNA were determined using a commercial quantitative RT-PCR kit (Kapa SYBR, Woburn, MA, USA). Reactions were subjected to 95°C for 2 min, followed by 40 cycles at 95°C for 30 s, 55°C for 20 s, and 72°C for 20 s. Each sample was performed in triplicate. Primer sequences were as follows: MAGEA12 Forward, 5ʹ-AGT CCG AGT TCC AAG CAG- 3ʹ; MAGEA12 Reverse, 5ʹ-TGA TGG TAG TGG GAA AGG-3ʹ; β-actin Forward, 5ʹ-GAT CAT TGC TCC TCC TGA GCA-3ʹ; and β-actin Reverse, 5ʹ-CAC CTT CAC CGT TCC AGT TTC-3ʹ. Levels of *MAGEA12* mRNA relative to those of β-actin were calculated using the 2^−ΔΔCt^ method.

### Assay of MAGEA12 and other protein levels

The western blotting assay was performed as previous study [22]. Briefly, cells were collected and lysed in RIPA buffer (Kapa SYBR), and total protein was fractionated by 10% SDS-PAGE and transferred to nitrocellulose membranes (Millipore, Shanghai, China). Membranes were blocked in 10% milk; incubated for 12 h at 4°C with primary antibodies (1:1000, Sigma-Aldrich, USA) against MAGEA12, Akt, phosphorylated Akt (p-Akt), cyclin D1, CDK14, or β-actin; and then incubated for 1.5 h with secondary antibody IgG conjugated with horseradish peroxidase (1:3000, Sigma-Aldrich, USA). The blots were incubated for 4 min in enhanced chemiluminescence reagent (Roche), and densitometry was performed using Image J software (National Institutes of Health, Bethesda, MD, USA). Band intensities were normalized to those for β-actin.

### Assay of cell viability

The CCK-8 assay was carried out as previous observation [23]. Briefly, at 24 h after transfection, Cal-27 cells were grown in 96-well plates at 37°C and cultured for 24 h. Then, 10 μL of CCK-8 solution was added to each well, and the plates were incubated another 2 h. Absorbance at 450 nm was measured.

### Assay of colony formation

At 24 h after transfection, Cal-27 cells were grown in 6-well plates at 37°C and cultured for 24 h. Cells in 10 fields of view were counted under an inverted microscope, and the average number was calculated.

### Statistical analysis

All experiments were repeated three times. Differences between groups were assessed for significance using Student’s *t* test, with *P* < 0.05 defined as significant.

## Results

In our study, we supposed that resveratrol could suppress the proliferation of OSCC cells by suppressing MAGEA12/Akt signaling pathway. To confirm the oncogenic effects of MAGEA12, we first observed the effects of its overexpression on the growth of OSCC cells and the expressions of p-Akt, Cyclin D1, and CDK14. Then, the potential roles of resveratrol in the growth of OSCC cells and MAGEA12/Akt signal transduction were explored.

### MAGEA12 overexpression induces the proliferation of OSCC cells

**Figure 1.**
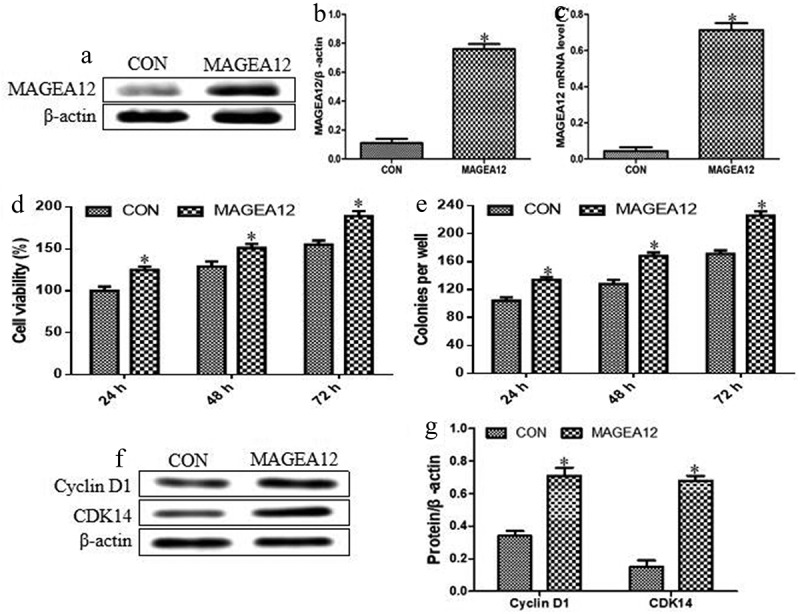
MAGEA12 overexpression induces proliferation and colony formation of OSCC cells. MAGEA12 plasmid was transfected into Cal-27 cells after 24 h, then protein and RNA were collected. Control cells were transfected with empty vector. (a) Western blot strips of MAGEA12 protein. (b) The relative expression levels of MAGEA12 protein by quantitation. (c) Quantitative RT-PCR of MAGEA12 mRNA. (d) Viability based on the CCK8 assay. (e) Colony formation based on analysis under an inverted microscope. The expression of Cyclin D1 and CDK14 was assessed by (f) western blot with (g) quantitation. Control cells were transfected with empty vector. *P < 0.05 vs control group (CON)

Cal-27 cells were transiently transfected with a plasmid encoding *MAGEA12*, and overexpression was confirmed at the mRNA and protein levels (Figure 1a-c). We found that the expression levels of MAGEA12 protein and mRNA were significantly increased in cells transfected with the plasmid encoding *MAGEA12* compared with control cells. These results suggested that MAGEA12 overexpressing cell model was successfully constructed.

Then, we further observed the effects of MAGEA12 overexpression on the proliferation of OSCC cells. MAGEA12 overexpression significantly increased the viability of Cal-27 cells, indicating that it promoted proliferation (Figure 1d). It also significantly increased colony formation (Figure 1e). Moreover, the inducible effects of MAGEA12 overexpression on the cell proliferation and colony formation were significantly increased compared with control cells with a time-dependent manner. These results demonstrated that MAGEA12 overexpression promotes the development of OSCC.

Additionally, the impacts of MAGEA12 overexpression on the expression levels of Cyclin D1 and CDK14 were also investigated by quantitating western blot bands of proteins. As shown in Figure 1f **and G**, MAGEA12 overexpression significantly up-regulated Cyclin D1 and CDK14. These results indicate that, indeed, MAGEA12 overexpression promoted the cell proliferation in OSCC.

### MAGEA12 induces proliferation of Cal-27 cells by activating Akt

**Figure 2.**
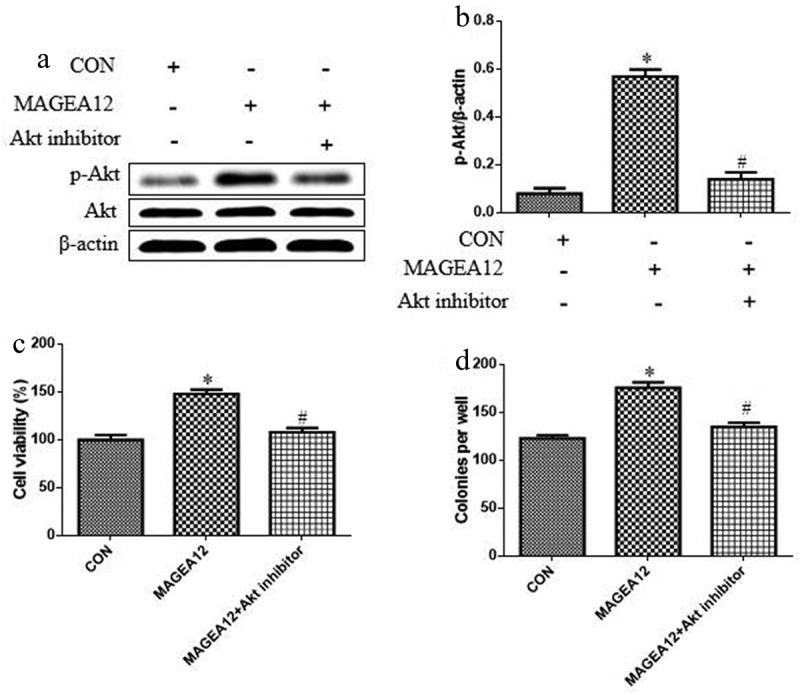
MAGEA12 promotes Akt signaling to induce growth of OSCC cells. MAGEA12 plasmid was transfected into Cal-27 cells for 24 h, and then cells were treated with Akt inhibitor MK-2206 (2 μM) for 12 h. Levels of Akt and p-Akt were (a) determined by western blot and (b) quantitated. Then assessed for (A) viability using the CCK8 assay and (B) colony formation. *P < 0.05 vs control group (CON). ^#^*P* < 0.05 vs MAGEA12 overexpression group (MAGEA12)

Given the involvement of the Akt cascade in tumorigenesis, proliferation, metastasis, invasion, and the EMT, we asked whether MAGEA12 may exert its proliferative effects by activating Akt. Indeed, MAGEA12 overexpression dramatically up-regulated levels of p-Akt, and this up-regulation was partially reversed in the presence of the Akt inhibitor MK-2206 (Figure 2a and b).

We also tested whether MAGEA12 overexpression acts via Akt signaling to induce growth of OSCC cells by treating MAGEA12-overexpressing cells with Akt inhibitor MK-2206, and then assaying cell viability and colony formation. Inhibiting Akt partially reversed the ability of MAGEA12 to promote proliferation and colony formation (Figure 2c and d), implying that Akt signaling helps mediate the oncogenic effects of MAGEA12.

### Resveratrol suppresses the cell growth and MAGEA12/Akt signaling pathway in OSCC cells

**Figure 3.**
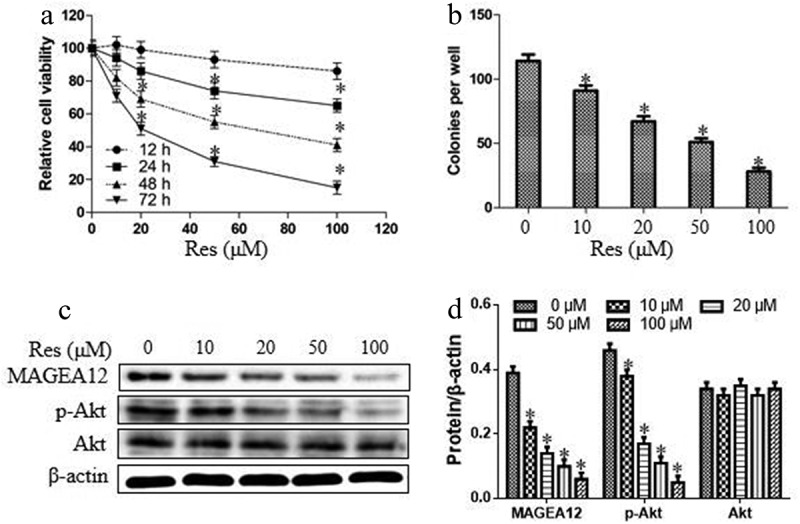
Resveratrol suppressed cell proliferation and MAGEA12/Akt cascade in OSCC cells. (a) The relative cell viability was detected by CCK8 assay after treatment with resveratrol in different concentrations and time points. (b) The numbers of cell colonies were measured by microscope. (c-d) Western blot and quantitation. Compared with 0 μM of resveratrol group, *P < 0.05

Cal-27 cells were treated with resveratrol in gradient of concentrations (0, 10, 20, 50, and 100 μM) and time points. As shown in Figure 3a, resveratrol inhibited the cell viability of Cal-27 cells depending on the time and concentration. These results suggested that the drug concentration-proliferation curve in time point of 48 h was acceptable, and the half maximal inhibitory concentration (IC_50_) of resveratrol was approximately 50 μM. Thus, in the subsequent tests, the function time and concentration were set 48 h and 50 μM, respectively. Furthermore, we observed the impacts of resveratrol the cell colony and MAGEA12/Akt cascade in OSCC cells. As shown in Figure 3b-d, resveratrol decreased the numbers of colonies and the expression levels of MAGEA12 and p-Akt. These findings suggest that resveratrol could suppress the cell growth and MAGEA12/Akt signaling pathway in OSCC cells.

### Resveratrol suppresses the cell growth and the activation of Akt in OSCC cells by suppressing MAGEA12

**Figure 4.**
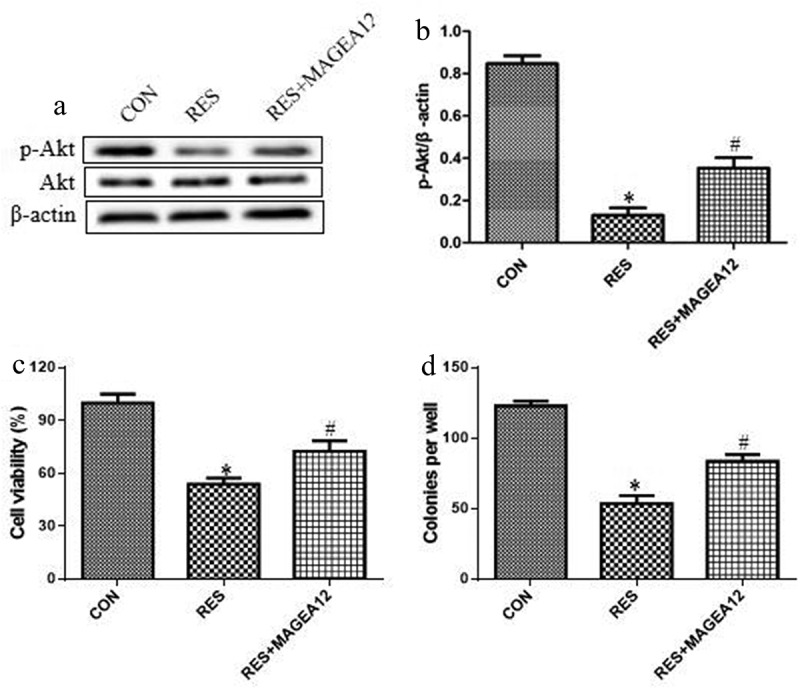
Resveratrol inhibits MAGEA12 signaling to decrease the levels of p-Akt and the growth of OSCC cells. MAGEA12-overexpressing cells were treated with resveratrol, then levels of Akt and p-Akt were determined by (a) western blot and (b) quantitated. Next we assessed for (c) cell viability using the CCK8 assay and (d) colony formation. *P < 0.05 vs control group (CON). ^#^*P* < 0.05 vs resveratrol group (RES)

We investigated whether resveratrol acts via MAGEA12 signaling to suppress the growth of OSCC cells and the activation of Akt by treating MAGEA12-overexpressing cells with resveratrol, and then determining cell viability and colony formation. MAGEA12 overexpression partially reversed the inhibitory effects of resveratrol on the phosphorylation levels of Akt (Figure 4a and b), suggesting that resveratrol inhibits p-Akt expression via decreasing MAGEA12. MAGEA12 overexpression partially reversed the inhibitory effects of resveratrol on proliferation and colony formation (Figure 4c and d), indicating that resveratrol inhibits the growth of OSCC cells by down-regulating MAGEA12.

### Resveratrol inhibits the growth of OSCC cells by down-regulating Akt activation

**Figure 5.**
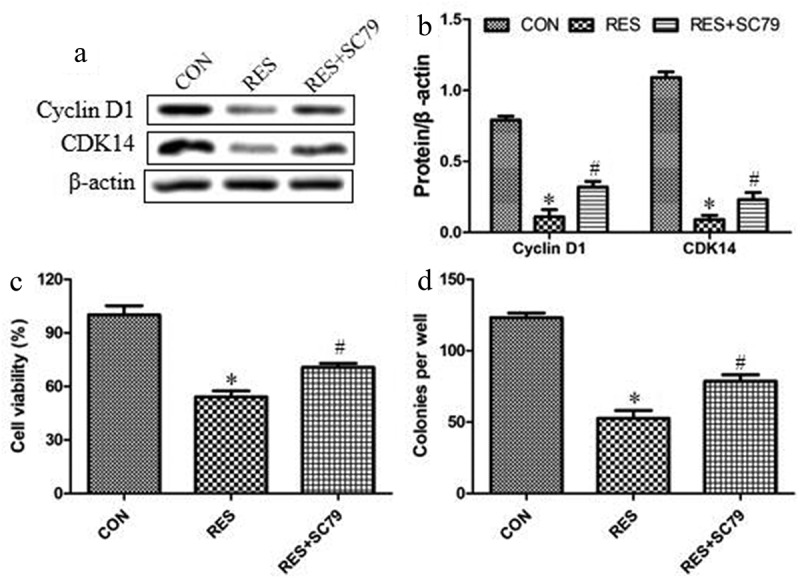
Resveratrol inhibits Akt signaling to decrease the levels of cyclin and the growth of OSCC cells. Cells were treated with resveratrol or Akt agonist SC79 (2 μM) for 24 h, then levels of Cyclin D1 and CDK14 were determined by (a) western blot and (b) quantitated. Next we assessed for (c) cell viability using the CCK8 assay and (d) colony formation. *P < 0.05 vs control group (CON). ^#^*P* < 0.05 vs resveratrol group (RES)

We investigated whether resveratrol acts via Akt signaling to suppress the growth of OSCC cells and the upregulation of Cyclin D1 and CDK14 by treating MAGEA12-overexpressing cells with resveratrol, and then determining cell viability and colony formation. Akt agonist (SC79) partially reversed the inhibitory effects of resveratrol on the expression levels of Cyclin D1 and CDK14 (Figure 5a and b), suggesting that resveratrol inhibits cell proliferation via decreasing Cyclin D1 and CDK14 expressions. SC79 also partially reversed the inhibitory effects of resveratrol on proliferation and colony formation (Figure 5c and d), indicating that resveratrol inhibits the growth of OSCC cells by down-regulating Akt activation.

## Discussion

It is well known that MAGE family members exert two types, including type I and type II MAGEs [24]. Type II MAGE members are always expressed in healthy tissues in humans, but not involved in the occurrence or development of cancers. Type I proteins in the melanoma-associated antigen gene family, including *MAGEA12*, are epigenetically silenced in healthy tissues except in the testes and placenta [25]. They are abnormally expressed in malignant tumors, making them attractive therapeutic targets [26–28]. MAGEA12 has been linked to the development or progression of prostatic carcinoma and colorectal cancer [7], as well as OSCC, gastric cancer, and lung cancer [29–31]. Here we demonstrate that MAGEA12 overexpression promotes the proliferation, colony formation and EMT in OSCC cells. Previous work has shown that Cyclin D1 and CDK14 contribute to cell cycle progression and regulate cells in a highly proliferative status [32]. Our study also found that MAGEA12 overexpression promoted the upregulation of Cyclin D1 and CDK14 and further induced the proliferation of OSCC cells. These findings are consistent with the previous report that MAGEA12 down-regulated the cyclin-dependent kinase inhibitors (p21) and involved in the pathogenesis of cutaneous squamous cell carcinoma [33]. We provide evidence that these oncogenic effects are mediated, at least in part, by activation of Akt signaling.

Previous study has verified that MAGEA12 regulates cell cycle via inducing p21 expression, which further involves in the activation of PI3K/Akt signaling pathway [34,35]. Thus, we speculated that MAGEA12 may promote the proliferation, colony formation, and EMT in OSCC cells via activating Akt target. In this study, we found that MAGEA12 overexpression induced the upregulation of Akt expression in Cal-27 cells, suggesting that MAGEA12 could positively regulate the signal transduction of Akt.

Extensive observations have reported that the upregulation of Akt contributes to the occurrence and development of multiple cancers, indicating that Akt is considered as a therapeutic target for tumors [36–38]. Therefore, Akt has been shown to function as an oncogene in cancer progression, including OSCC [39,40]. Recent studies aimed to explore the Akt toward the molecular targeted treatment using small-molecule inhibitors in human tumors. Although it is unclear that whether MAGEA12 could regulate the Akt expression in OSCC cells, several evidence have shown that the indirect association between MAGEA12 and Akt was found [8,16]. We further confirmed that whether MAGEA12 promotes the proliferation, colony formation, and EMT of Cal-27 cells through the regulation of Akt. Thus, the Akt inhibitor MK-2206 was used to incubate the cancer cells transfected with MAGEA12 plasmid. And our data showed that Akt inhibition partially reversed the ability of MAGEA12 to promote proliferation and colony formation, implying that Akt signaling helps mediate the oncogenic effects of MAGEA12.

We further observed the effects of resveratrol on the viability, colony and MAGEA12/Akt pathway in OSCC cells. Many studies have reported that resveratrol exerts antiproliferative effect and induction of apoptosis in various tumor cells [41,42]. Furthermore, resveratrol has been demonstrated to overcome cetuximab-resistance in OSCC by targeting urokinase-type plasminogen activator receptor [43]. Additionally, extensive observations have also reported that resveratrol suppressed the growth of OSCC cells by regulating chromobox protein homolog 7 (CBX7) signal transduction, inducing mitochondrial apoptosis and G2/M phase cell cycle arrest, and inhibiting epithelial–mesenchymal transition [44–46]. However, the association among resveratrol and MAGEA12 has not been reported. Therefore, our results superficially suggested that resveratrol acts via MAGEA12/Akt signaling to inhibit the proliferation of OSCC cells. Whereas the detailed mechanism of resveratrol in the inhibition of MAGEA12 protein expression remains unclear, and we will further explore it in vitro.

### Conclusion

Taken together, our results should be verified and extended in other OSCC cell lines and in preclinical models. MAGEA12 may be a useful target for treating this disease.

## Highlights

- MAGEA12 induces OSCC cells growth.
- MAGEA12 activates Akt signaling.
- Resveratrol suppresses the proliferation of OSCC cells via MAGEA12/Akt pathway.
